# Electroosmotic Flow Hysteresis for Fluids with Dissimilar pH and Ionic Species

**DOI:** 10.3390/mi12091031

**Published:** 2021-08-28

**Authors:** An Eng Lim, Yee Cheong Lam

**Affiliations:** School of Mechanical and Aerospace Engineering, Nanyang Technological University, 50 Nanyang Avenue, Singapore 639798, Singapore; lima0028@e.ntu.edu.sg

**Keywords:** micro-/nanofluidics, electrokinetic phenomena, electroosmotic flow hysteresis, current monitoring method, numerical simulation

## Abstract

Electroosmotic flow (EOF) involving displacement of multiple fluids is employed in micro-/nanofluidic applications. There are existing investigations on EOF hysteresis, i.e., flow direction-dependent behavior. However, none so far have studied the solution pair system of dissimilar ionic species with substantial pH difference. They exhibit complicated hysteretic phenomena. In this study, we investigate the EOF of sodium bicarbonate (NaHCO_3_, alkaline) and sodium chloride (NaCl, slightly acidic) solution pair via current monitoring technique. A developed slip velocity model with a modified wall condition is implemented with finite element simulations. Quantitative agreements between experimental and simulation results are obtained. Concentration evolutions of NaHCO_3_–NaCl follow the dissimilar anion species system. When NaCl displaces NaHCO_3_, EOF reduces due to the displacement of NaHCO_3_ with high pH (high absolute zeta potential). Consequently, NaCl is not fully displaced into the microchannel. When NaHCO_3_ displaces NaCl, NaHCO_3_ cannot displace into the microchannel as NaCl with low pH (low absolute zeta potential) produces slow EOF. These behaviors are independent of the applied electric field. However, complete displacement tends to be achieved by lowering the NaCl concentration, i.e., increasing its zeta potential. In contrast, the NaHCO_3_ concentration has little impact on the displacement process. These findings enhance the understanding of EOF involving solutions with dissimilar pH and ion species.

## 1. Introduction

An applied electric field induces electroosmotic flow (EOF) of fluids in a micro-/nanochannel. Upon contact with an aqueous solution, the negative charges developed on the channel wall result in the repulsion and attraction of the negative and positive ions in the electrolyte solution. Electrical double layer (EDL) of nanometer thickness is formed as a result of the net positively charged layer. When an electric field is applied, an electrical body force is experienced by the EDL, which drives its motion along the direction of the electric field. Through viscous drag, the bulk fluid acquires the flow momentum from the EDL. This generates a plug-like fluid flow. With comparatively thin EDL thickness to channel dimensions, the Helmholtz-Smoluchowski equation can be used to determine the EOF velocity:

*V_EOF_* = *−ε_r_ε_o_ζE*/*µ*, (1)

where *ε_r_* and *ε_o_* are the fluid relative permittivity and the free space permittivity, respectively, *µ* is the fluid viscosity, *E* is the electric field applied externally, and *ζ* is the electrostatic wall zeta potential. The EDL thickness (for a symmetric electrolyte) can be characterized by the Debye length:

*λ_D_* = (*ε_r_ε_o_k_b_T*/2*z*^2^*e*^2^*N_a_c_o_*)^1/2^, (2)

where *k_b_* is the Boltzmann constant, *N_a_* is the Avogadro constant, *e* is the electron charge, *T* is the temperature, *c_o_* is the electrolyte solution concentration, and *z* is the ion species charge number.

Electroosmotic flow (EOF) involving displacement of multiple fluids is widely employed in micro-/nanofluidic applications. These include electrokinetically controlled DNA hybridization [1,2,3], analyte mixing [4,5], fluid pumping [6,7,8,9], chemical species/particle separation [10], and pre-concentration [11,12]. In micro-energy systems, liquid flooding is a typical problem in the Proton Exchange Membrane (PEM) fuel cell, which reduces the fuel cell power greatly. With the integration of planar electroosmotic pump (EOP), the excess liquid is actively removed through microflow channels [7]. Mixing of analytes can be accomplished with the electrokinetic instability (EKI) approach by rapid folding and stretching of fluids through proper designs of micro-mixing devices [4].

In numerous practical applications, multiple fluids with dissimilar properties, e.g., concentration and conductivity, are commonly involved. For instance, pre-concentration methods such as isotachophoresis (ITP) and field amplified sample stacking (FASS) [11,12] involve micro-mixing of fluids with dissimilar conductivities. With a constant current setting, ITP forms zones of concentrated/focused sample ions in order of the ionic mobility [13,14,15]. In contrast, FASS employs an applied electric field with a constant voltage to stack/accumulate the sample ions at the interface between the low conductivity sample solution and the high conductivity background electrolyte (BGE) [16,17]. Sample dispersion due to the non-uniform EOF velocities adversely affects the resolution and sensitivity of both ITP and FASS; this can be minimized by coating water-soluble polymers [18,19], and constructing nanostructures within a microchannel [20,21,22].

Many investigations have been conducted on EOF of fluids with different conductivities/concentrations [23,24,25,26]. Tang et al. [23] performed an investigation on electroosmotic displacement flow with two or three fluids of different conductivity ratios, with the velocity of the fluid interface and the current-time response theoretically derived. Ren et al. [24,25] conducted two-fluid displacement flow experiments for various solution concentrations. They related the flow processes with the electrical equivalent circuit model, whereby the fluid resistances were determined by their electrical conductivities. Mampallil et al. [26] adopted two-fluid electroosmotic displacement flow experiments to measure the surface charge of microchannel. The current-time response of the displacement process was curve-fitted to a theoretical expression to acquire the surface charge measurement. In addition, there are also numerous applications that involve EOF of several fluids, such as ionic transistor and diode [27,28], and transdermal drug delivery [29]. These examples [23,24,25,26,27,28,29] have demonstrated the importance of EOF on multiple fluid systems in practical applications.

However, the EOF of two dissimilar fluids demonstrates the hysteresis effect experimentally, known as EOF hysteresis, as coined by our group [30,31,32,33], whereby the EOF velocity/flow rate for fluid 1 displacing fluid 2 is different from fluid 2 displacing fluid 1. The hysteretic behavior is exhibited as the EOF velocity/flow rate and the final content in a microchannel are influenced by the interplay between the electric field distribution, induced EOF flow and ion migration; these are functions of the direction of displacement flow. Understanding the effect of EOF hysteresis is essential as its neglection may result in experimental data to be interpreted inappropriately.

Our group have numerically investigated EOF hysteresis involving fluids with different concentrations [30]. Rather counter-intuitively, it was discovered that the hysteresis effect arises from the depletion and accumulation of the minority pH-governing ions caused by the electromigrative flux imbalance; the pH, zeta potential, and EOF velocity are altered differently according to the displacement flow directions. The mechanics have been affirmed by the investigation of Li et al. [34]. By including the proton transport, carbonate equilibrium, and surface complexation reaction equations, their semi-analytical model can capture the experimentally observed hysteretic effect. Further study had been performed by our group to validate the hypothesis [31]. With the application of pH-sensitive dye, the pH changes in the microchannel during EOF for different displacement flow directions were experimentally quantified. 

We had also investigated EOF hysteresis for fluids with dissimilar ionic species [32,33]. Two mechanics were discovered to induce EOF hysteresis for fluids with dissimilar cation species [32], namely the interfacial sharpening/widening effect caused by the difference in solution conductivities, and the ion concentration evolutions, that lead to the variation of EOF velocities/flow rates for different flow directions. Contrary to conventional electroosmotic displacement flows, the EOF of fluids with dissimilar anion species [33] was found to illustrate complex behavior, i.e., the displacing fluids cannot fully displace the residing fluids. The observed EOF hysteresis is caused by the changing ion concentrations due to the upstream migrating anions, and the diffusive-interface-induced concentration changes at the inlet of microchannel.

Despite previous studies [30,31,32,33], no investigation has heretofore been carried out on EOF hysteresis for a solution pair system comprising of dissimilar ionic species and substantial pH difference. Their rather different characteristics tend to result in a complex hysteretic behavior, thus rendering a more complex analysis. Our previous simulation models [30,31,32,33] will require excessive computational effort for this investigation, and thus may not be able to accurately reflect the displacement flow conditions due to computational constraint. Therefore, a slip-velocity model with modified wall condition is developed, which could describe the phenomena exhibited with sufficient precision and accuracy as compared to our previous models [30,31,32,33], but with much less computational effort.

A comprehensive understanding of their hysteresis phenomena is necessary to provide accurate EOF interpretation for analytical systems [35,36,37] involving fluids with dissimilar pH and ionic species. The findings of this investigation will aid in the precise manipulation of EOF conditions for effective improvement in micro-/nanofluidic applications with inhomogeneous solutions.

## 2. Experimental Methods and Materials

### 2.1. Experimental Parameters and Conditions

Investigation of EOF hysteresis for a solution pair system comprising of dissimilar ionic species and substantial pH difference was conducted. Sodium bicarbonate (NaHCO_3_) and sodium chloride (NaCl) solution pair was selected as a model for the examination of the hysteresis behavior. NaHCO_3_ alkaline salt breaks down to form sodium ion Na^+^ and bicarbonate ion HCO_3_^−^ in water, resulting in an alkaline solution (high pH). While NaCl neutral salt breaks down to form Na^+^ and chloride ion Cl^−^ in water, which gives a slightly acidic solution (low pH) due to the dissolved carbon dioxide from the atmosphere [30,31]. The characteristics of the solution pair complicate the electroosmotic displacement flow process. The solution concentrations and applied electric field were varied to comprehend their effects on the hysteresis phenomena. The experimental conditions and parameters investigated are shown in Table 1.

A numerical investigation was conducted to study the effects of varying the concentration of the solution pair and applied electric field (see Section 3). Knowledge gained from understanding these systems with reference to the basic fluid configuration (i.e., same concentration for NaHCO_3_ and NaCl solutions, see Table 1) was employed in the exploration of the underlying mechanics that influence the displacement process, when the concentration of each individual solution was varied.

### 2.2. Experimental Setup and Materials

**Figure 1:**
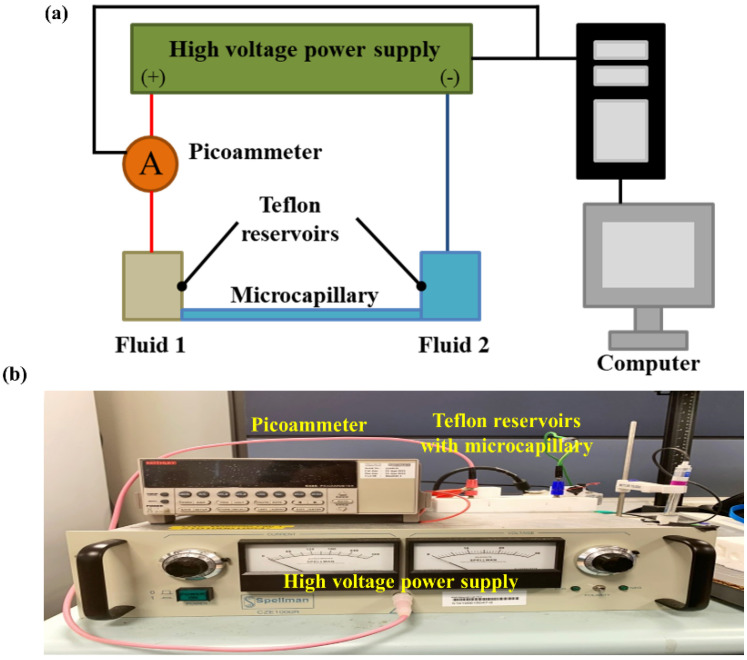
(**a**) Schematic for current-time monitoring setup, with (**b**) actual experimental setup.

Current monitoring technique [30,31,32,33] was employed for the observation of EOF behavior during displacement flow process (see Section 2.3), and the measurement of the zeta potential (see Section 2.4). An illustration of the experiment setup is depicted in Figure 1. A high voltage power supply (CZE1000R, Spellman, Hauppauge, NY, USA) was used to supply the electric field for inducing EOF. To monitor the current change across the microchannel, a picoammeter (Keithley 6485, Tektronix, Singapore) was connected in series. The two devices were controlled by a customized Labview program to record the readings of current and voltage with a data acquisition card (PCI-6052E, National Instrument, Austin, TX, USA).

By dissolving the NaHCO_3_ and NaCl salts (Sigma-Aldrich, Saint Louis, MO, USA) in deionized (DI) water, 0.01 M stock solutions for NaHCO_3_ and NaCl were prepared. The prepared solutions were further diluted to the concentrations required for experimentation (see Section 2.1). Measurements of the solution properties, i.e., pH and conductivity, were carried out with a pH meter (Mettler Toledo, FiveEasy plus, Singapore) and conductivity meter (IONCheck 65, Radiometer Analytical, Loveland, CO, USA). Table 2 presents the measured pH values and conductivities.

Microchannels, i.e., flexible fused silica microcapillaries (polyimide coated, Polymicro Technologies, Phoenix, AZ, USA), with 100 µm nominal internal diameter were cut into 8 cm length with a Shortix Column Cutter (SGT Ltd., Singapore). Acetone was used to flush the microcapillaries, followed by DI water. Lastly, the capillaries were filled with the electrolyte solutions. The two ends of the microchannel were connected to two reservoirs made of Telfon having both depth and diameter of 2 cm (see Figure 1). 

The zeta potential developed at the glass/silica surface is caused by the deprononation (proton removal) of silanol (SiOH) groups in contact with an aqueous solution: SiOH + H_2_O ⇌ SiO^−^ + H_3_O^+^ [30]. For a decrease in pH, i.e., an increase in H_3_O^+^ concentration, the equilibrium will shift to the left, resulting in less SiO^−^ groups; this lower concentration of the negatively-charged groups on the glass surface will reduce the absolute zeta potential value. The reverse is true for an increase in pH.

Upon contact with the NaCl or NaHCO_3_ electrolyte solutions, the negative charges developed on the channel wall result in the repulsion of the Cl^−^ or HCO_3_^−^ (negative) and attraction of the Na^+^ (positive) ions. This forms a net positively charged EDL layer. When an electric field is applied, an electrical body force is experienced by the EDL, which drives its motion along the direction of the electric field. Through viscous drag, the bulk fluid acquires the flow momentum from the EDL, which generates a plug-like fluid flow. The ion mobilities of Na^+^, Cl^−^ and HCO_3_^−^ are 5.194 × 10^−8^ m^2^·V^−1^·s^−1^, −7.919 × 10^−8^ m^2^·V^−1^·s^−1^ and −4.303 × 10^−8^ m^2^·V^−1^·s^−1^, respectively. Based on the ion mobilities, the ion transport numbers, defined as the fractions of the total electrical current carried by the ion species, can be calculated. For NaCl solution, the ion transport numbers of Na^+^ and Cl^−^ are 0.396 and 0.604, respectively; for NaHCO_3_ solution, the ion transport numbers of Na^+^ and HCO_3_^−^ are 0.547 and 0.453, respectively.

As the size of the reservoirs was sufficiently large, the change in liquid level during the experiment was negligible. This minimized the back pressure from the liquid level difference in the reservoirs [38]. With small reservoirs, EOF might be affected by the local pH change induced by electrolysis at the electrodes due to the generation of H^+^ and OH^−^ ions [39]. The use of large-volume reservoirs significantly diluted the H^+^ and OH^−^ concentrations. The electrodes were also placed distant from the microchannel inlet and outlet (approximately 2 cm) to avoid unnecessary change in pH in the channel [40].

### 2.3. EOF of NaHCO_3_–NaCl Solution Pair System

Electroosmotic displacement flow experiments for the NaHCO_3_–NaCl solution pair system were performed via current monitoring technique (see Figure 1), according to the experimental parameters and conditions in Table 1. The experiments were conducted in two displacement flow directions, i.e., NaCl → NaHCO_3_ (the arrow indicates EOF direction), and NaHCO_3_ → NaCl. The displacing solution was placed in the anode reservoir for displacing the original residing solution in the microchannel; the channel and cathode reservoir were filled with the residing solution. For the revelation of the hysteresis behavior, comparisons of the current-time responses in two opposite flow directions were made. 

Due to the low conductivities and concentrations of the electrolytes in this investigation, the effect of joule heating is negligible [41]. By considering the balance in energy between the energy storage Δ*E_st_* and energy generation *E_g_* in the fluid, the effect of joule heating can be conservatively estimated with the methodology detailed in the work of Arulanandam and Li [42]. The ion concentrations will be changing during the experiments. Hence, the worst scenario was having NaHCO_3_ concentration = 5 mM, microchannel cross-sectional area = 7850 µm^2^, applied electric field = 125 V·cm^−1^ and experimental duration = 600 s; the estimated temperature increase is only 0.8 °C and is insignificant to affect the electroosmotic displacement flow process.

### 2.4. Zeta Potential Measurements

Zeta potential is a crucial parameter which determines the EOF velocity for electroosmotic displacement flow in a microchannel. As numerical simulations are required for the evaluation of the displacement flow behaviors and ion concentration distributions, it will be necessary for the prescription of exact wall zeta potential conditions to obtain accurate EOF simulations (see Section 3). Therefore, zeta potential measurements of the solutions required for simulations will be carried out.

Current monitoring technique (see Figure 1) was employed for the measurement of zeta potential. The cathode reservoir and microchannel were filled with the solution for measurement (residing solution), while the anode reservoir was filled with 95% concentration of the measurement solution (displacing solution), i.e., 5% difference in concentration. Electric field of 125 V·cm^−1^ was applied to generate EOF across the two reservoirs. To ensure reliability and consistency of the results, for each data point, the experiments were performed five times.

**Figure 2:**
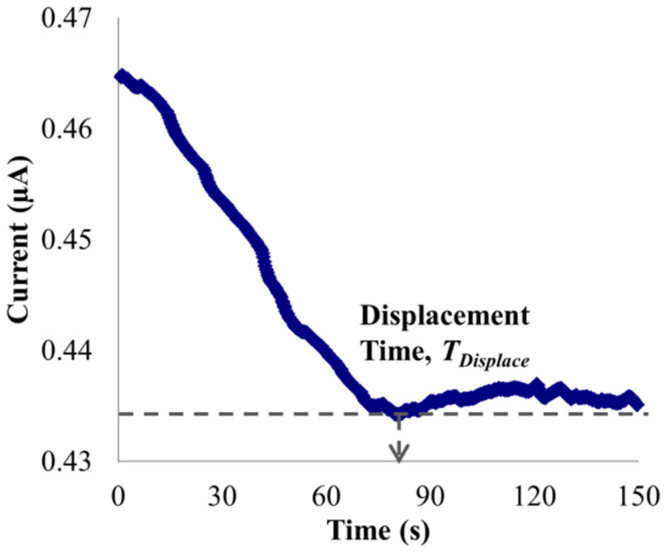
Current-time response for 0.475 mM NaHCO_3_ (95% concentration of the measurement solution) displacing 0.5 mM NaHCO_3_ (measurement solution).

Through dividing the microchannel length with the displacement time, i.e., time for the current to attain the steady-state value of displacing solution (see Figure 2), the EOF velocity can be calculated with [30,31,32,33]:

*V_EOF_* = *L*/*T_Displace_*, (3)

where *L* and *T_Displace_* represent the microchannel length and the displacement time, respectively.

Thereafter, by substituting the measured EOF velocity from Equation (3) into the Helmholtz-Smoluchowski slip velocity equation (see Equation (1)), the zeta potential may be written as [30,31,32,33]:

*ζ* = −*μL*/(*ε_r_ε_o_ET_Displace_*), (4)

where *μ* is the fluid viscosity, *ε_r_* and *ε_o_* are the fluid relative permittivity and the free space permittivity, respectively, and *E* is the applied electric field.

The measured zeta potential values of the solutions required for the numerical simulations (see Section 3) are shown in Table 3. Table 3 indicates that by lowering the NaCl concentration from 1.24 mM to 0.5 mM, i.e., by approximately 60%, the absolute zeta potential value increased significantly by about 40%. By decreasing the NaCl concentration, there will be lesser Na^+^ ions (positive ions) for the shielding of the negative wall charge (fused silica), which leads to the increase of absolute zeta potential value. While lowering the NaHCO_3_ concentration from 1 mM to 0.375 mM, i.e., by approximately 60%, the absolute zeta potential value increased only by about 10%, i.e., much less. The absolute zeta potential value is supposed to similarly increase significantly when the NaHCO_3_ concentration is decreased. However, decreasing the NaHCO_3_ concentration causes the pH to decrease (see Table 2), which will cause a decrease in the absolute zeta potential value. The competing effect between the solution concentration and pH results in the slight increase of the absolute zeta potential value for the case of NaHCO_3_.

## 3. Numerical Simulations

### 3.1. Numerical Model

Conventional theoretical models, i.e., slip-velocity [43,44,45,46] and Poisson-Boltzmann (PB) models [47,48], mainly describe a single fluid EOF by prescribing a constant zeta potential along the microchannel. However, the zeta potential and ion concentration distributions are expected to vary during electroosmotic displacement flow [30,31,32,33]. Hence, these models cannot adequately describe the displacement flow process.

The Poisson-Nernst-Planck (PNP) model [32,33] considers the electric field variation (due to a difference in solution conductivity) and the transportation of main constituent ionic species during two-fluid displacement flow. With its ability to capture the transportation of the ionic species under diffusive, convective, and electromigrative effects, the variation of zeta potential is accounted for by prescribing a constant surface charge density at the microchannel wall.

Surface charge regulation model [30,49] provides a better elucidation of the two-fluid displacement flow process. In addition to the features of PNP model, the surface charge regulation model includes the transportation of minority pH-governing ionic species with its associated reversible acid-based chemical reactions. The model can simulate the variation of zeta potential due to the concentration changes of main constituent ionic species, and the pH changes attributed by the accumulation/depletion of minority pH-governing ionic species.

However, excessive computational effort will be required to implement the surface charge regulation model. Our investigation employs the NaHCO_3_–NaCl solution pair system, where dissimilar ionic species and substantial pH difference are involved, thus rendering a more complex analysis. However, the PNP model is not applicable for our study. This is because the large pH difference between the NaHCO_3_ and NaCl solutions (see Table 2) yields a large surface charge difference; as such, the specification of an average constant charge density will not accurately reflect the displacement flow conditions.

Therefore, a slip-velocity model with modified wall condition, which could describe the phenomena exhibited, will be adopted. This model, which has the same capabilities as the PNP model, considers the electric field variation (due to the difference in solution conductivity) and transportation of main constituent ionic species during two-fluid displacement flow. By prescribing a varying wall zeta potential boundary according to the flow condition, it can execute simulation runs with sufficient precision and accuracy as compared to the surface charge regulation model, but with much lesser computational effort.

### 3.2. Governing Equations

The modified slip-velocity model is developed based on several fundamental equations [30,31,32,33]. The applied electric potential *φ* is governed by the Laplace equation, which accounts for the electric field variation:

∇·(*σ*∇*φ*) = 0, (5)

where *σ = F∑z_i_u_m(i)_c_i_* is the solution conductivity, *c_i_*, *z_i_*, and *u_m(i)_* are the ion concentration, ion charge number, and ionic mobility, respectively, and *F* is the Faraday constant.

Governed by the gradients of diffusive, convective, and electromigrative fluxes respectively, the change of ionic species concentration with time is described by the Nernst-Planck (NP) equation:

*∂c_i_*/*∂t* + ∇[−*D_i_*∇*c_i_* − *u_m(i)_c_i_*∇*φ*] = −***v***·∇*c_i_*, (6)

where ***v*** is the velocity of the fluid, and *D_i_* the diffusion coefficient of the ion.

The flow of an incompressible Newtonian fluid is defined by the Navier-Stokes (NS) and continuity equations:

*ρ∂**v***/*∂t* = −∇*p* + *µ*∇^2^***v*** + *ρ_e_*[−∇(*φ*)], (7)

∇·***v*** = 0. (8)

where *p* is the pressure, *ρ* the density, and *µ* the fluid viscosity. As the Reynolds number is usually below 1 for a microchannel, the inertial term is to be neglected by assuming Stokes flow. 

The flow field is obtained by simultaneously solving the Laplace (Equation (5)), NP (Equation (6)), NS and continuity equations (Equations (7) and (8)). The values of the constants employed for the simulations can be found in Table 4.

### 3.3. Simulation Domain

**Figure 3:**
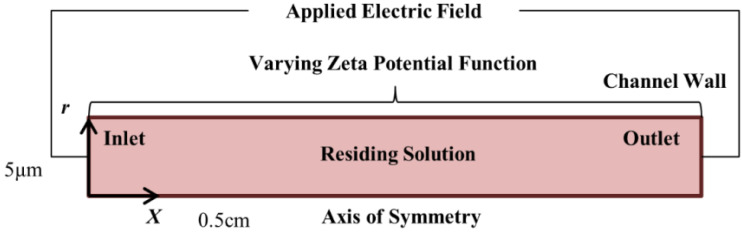
Domain for numerical simulation (not drawn to scale).

Finite element numerical simulations were performed using the software COMSOL Multiphysics. The simulated domain (see Figure 3) is a 10 μm diameter microchannel of 0.5 cm in length. Axisymmetry is assumed for the fluid flow about the center axis. The strong coupling between the applied electric potential, concentrations of ions and fluid flow velocity in the governing equations (see Section 3.2) will result in significant computational workload. To ease this workload, the domain diameter and length were reduced from 100 μm (experimentally) to 10 μm (numerically), and from 8 cm (experimentally) to 0.5 cm (numerically), respectively. For non-overlapping EDL, EOF will be independent of the microchannel diameter. Thus, the reduction of the microchannel diameter in the simulations will not affect the EOF displacement process. In addition, with the application of the same electric fields for both experiments and simulations, the reduction of the channel length in the simulations should not affect its accuracy in representing the flow behaviors of the experimental runs after normalization.

The simulated domain consisted of 24,000 quadrilateral elements (1000 and 24 edge elements, respectively, in the axial and radial directions). The fluid flow velocity and pressure were discretized with linear elements. While the ion concentrations and applied electric potential were discretized with quadratic elements. Through steady-state simulation, a convergence test was carried out with a higher element number, i.e., 30,000 elements (25% increment). The results were found to have a percentage change less than 1%, which is negligible. Between subsequent iterations, a relative tolerance of less than 0.01 (i.e., approximately 1%) was used as the convergence criterion.

### 3.4. Boundary and Initial Conditions

The steady-state solution for EOF of a single fluid is first obtained; it is used as the initial condition for solving the time-dependent solution of two-fluid electroosmotic displacement flow. The boundary conditions for the steady-state single fluid EOF numerical model are listed in Table 5.

The effects of varying the concentration of the NaHCO_3_–NaCl solution pair and applied electric field will be numerically studied. Ion concentrations of the simulation domain, inlet, and outlet (see Figure 3) were set according to the experimental conditions (see Table 1). Electroneutrality condition was enforced to satisfy charge neutrality. Voltages of 62.5 V and 93.75 V were prescribed at the inlet (0 V at outlet) to establish electric fields of 125 V·cm^−1^ and 187.5 V·cm^−1^, respectively. Electrical insulation condition was set at the microchannel wall to limit current flow within the channel. 

EOF of solutions with dissimilar anion species exhibits concentration evolutions [33]. The NaHCO_3_–NaCl solution pair consists of dissimilar anions Cl^−^ and HCO_3_^−^, and common cation Na^+^. Hence, concentration evolutions occur for displacement flow of the NaHCO_3_–NaCl solution pair, which follow the dissimilar anion species system. The evolved concentrations were determined from simulations, which can be found in Table 6. Knowing the evolved concentrations ensures that precise wall boundary conditions can be prescribed (see Table 7) for accurate EOF simulations.

Varying zeta potential functions (see Table 7) were specified at the wall boundary to capture the changes of zeta potential for different displacement flow directions. A formulation of these functions were based on the experimentally measured zeta potentials of the displacing, residing, and evolved concentration solutions for the various experimental flow conditions (see Table 1, Table 3 and Table 6). Based on these experimentally measured zeta potentials, these functions can accurately reflect the changes of zeta potential according to the content in the microchannel depending on the displacement flow conditions. The inlet and outlet pressures were prescribed to be zero.

The time-dependent electroosmotic displacement flow was solved by modifying the boundary conditions at the inlet. With the steady-state solution for EOF of a single fluid set as the initial condition, ion concentrations at the inlet were ramped from the residing solution to the displacing solution (see Table 1) in an arbitrarily short time of 0.01 s for commencing the displacement process.

## 4. Results and Discussion

**Figure 4:**
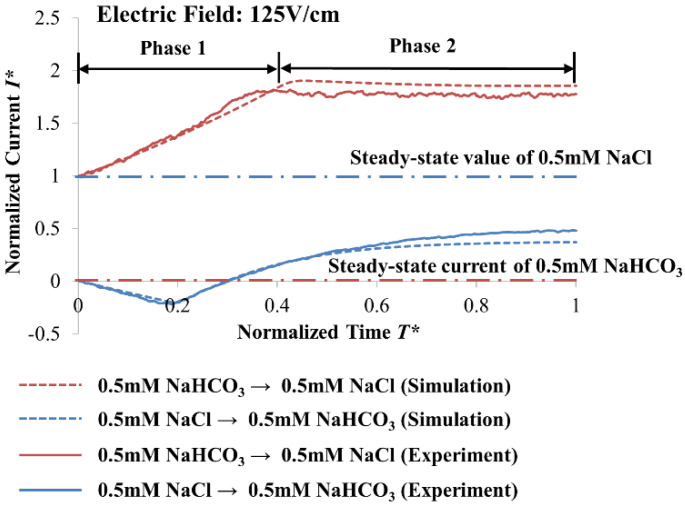
Electroosmotic displacement flow of the 0.5 mM NaCl–NaHCO_3_ solution pair at the applied electric field of 125 V·cm^−1^, whereby numerical simulation and experimental results are compared. Normalized currents and times are calculated with I* = (*I* − *I*^0^_*NaHCO*3_)/(*I*^0^_*NaCl*_ − *I*^0^_*NaHCO*3_) and T* = *T*/*T^SS^_NaCl_*_→ *NaHCO*3_ respectively, where initial currents of solutions are represented by *I*^0^ with *NaHCO*_3_ and *NaCl* subscripts respectively, and time to reach steady-state current for NaCl → NaHCO_3_ (arrow indicates EOF direction) is represented by *T^SS^_NaCl_*_→ *NaHCO*3_.

EOF behavior of the 0.5 mM NaHCO_3_–NaCl solution pair at the electric field of 125 V·cm^−1^ is shown in Figure 4. Normalization of the currents and times enables the comparison between the experimental and numerical simulation results. The normalized currents and times are calculated with Equations (9) and (10), respectively:

I* = (*I* − *I*^0^_*NaHCO*3_)/(*I*^0^_*NaCl*_ − *I*^0^_*NaHCO*3_) (9)

T* = *T*/*T^SS^_NaCl_*_→ *NaHCO*3_ (10)

where the initial currents of solutions are represented by *I*^0^ with *NaHCO_3_* and *NaCl* subscripts, respectively, and the time to reach steady-state current for NaCl → NaHCO_3_ (arrow indicates EOF direction) is represented by *T^SS^_NaCl_*_→ *NaHCO*3_.

Our slip-velocity EOF model with modified wall boundary condition for zeta potential variation (see Section 3) predicts the experimental observations excellently (see Figure 4). With the ability to facilitate accurate zeta potential variation to accommodate the displacement and evolution of the solution concentration, our numerical model can capture the essence of the experimental phenomena, and its validity is affirmed.

From the normalized current-time curves (see Figure 4), two phases were observed (namely Phases 1 and 2) to be separated by an abrupt gradient change, which typically occurs for the EOF of solutions with dissimilar ionic species [32,33]. For NaCl → NaHCO_3_ (the arrow indicates the EOF direction), a decrease and an increase in the current were observed for Phases 1 and 2, respectively (see Figure 4). In contrast, for 0.5 mM NaHCO_3_ → 0.5 mM NaCl, an increase and a decrease in current were observed for Phases 1 and 2, respectively (see Figure 4). For both cases, the current-time curves were unable to attain the steady-state values of the displacing solutions at the end of Phase 2, indicating that the displacing solutions were unable to displace completely the residing solutions.

**Figure 5:**
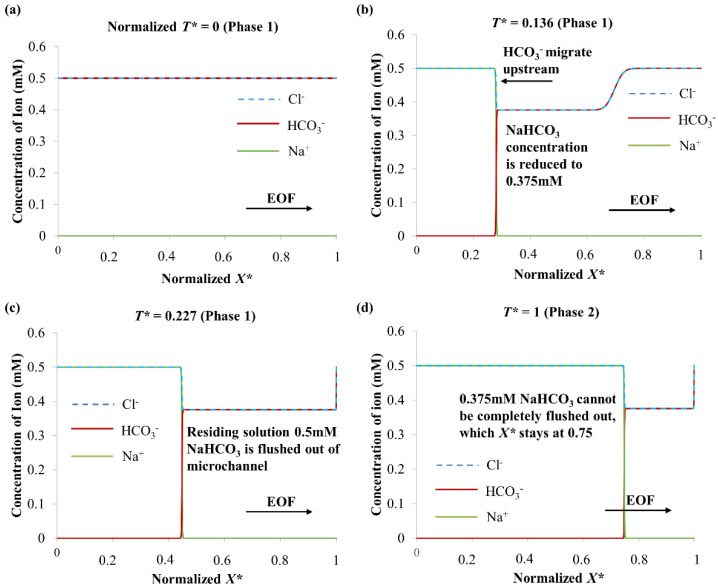
Numerically simulated ion concentrations along microchannel for 0.5 mM NaCl → 0.5 mM NaHCO_3_ (the arrow indicates EOF direction) in (**a**) Phase 1 T* = 0, (**b**) Phase 1 T* = 0.136, (**c**) Phase 1 T* = 0.227 and (**d**) Phase 2 T* = 1. Normalized time T* = *T*/*T^SS^_NaCl_*_→* NaHCO*3_, where *T^SS^_NaCl_*_→ *NaHCO*3_ is time to reach steady-state current for NaCl → NaHCO_3_. Normalized X* = X/*L*, where *X* is axial coordinate and *L* microchannel length.

Figure 5 shows the simulated evolution of ion concentration distributions for 0.5 mM NaCl → 0.5 mM NaHCO_3_. At normalized time T* = 0.136 (Phase 1), depletion of Na^+^ and HCO_3_^−^ ions occurs, with NaHCO_3_ concentration reducing to 0.375 mM. The change in concentration can be predicted by the Kohlrausch regulating function (KRF) [33,51], which satisfies the current continuity and electroneutrality:

KRF(*X*) = ∑[*z_i_c_i_*(*X,T*)/*u_m_*_(*i*)_] (11)

where *z_i_* is the ion charge number, *c_i_* the ion concentration as a function of microchannel axial coordinate *X* and time *T*, and *u_m_*_(*i*)_ the ion mobility.

An amount of 0.5 mM NaCl has a KRF value of 1.59 × 10^7^ mol·V·s·m^−5^, and 0.5 mM NaHCO_3_ has a KRF value of 2.12 × 10^7^ mol·V·s·m^−5^. Anions migrate upstream in opposition to the EOF flow. When 0.5 mM NaCl → 0.5 mM NaHCO_3_, the migration of HCO_3_^−^ causes a change in NaHCO_3_ concentration (see Figure 5b). The NaHCO_3_ concentration is lowered from 0.5 mM to 0.37 5mM to match the KRF of 0.5 mM NaCl (solution concentration derived from known KRF of 0.5 mM NaCl and ion mobilities of Na^+^ and HCO_3_^−^ from Table 4, see Equation (11)), with a corresponding drop in conductivity (see Table 2). Therefore, a decrease in current was observed during Phase 1 for 0.5 mM NaCl → 0.5 mM NaHCO_3_ (see Figure 4). When the residing solution 0.5 mM NaHCO_3_ was flushed out of the microchannel (at T* = 0.227 (Phase 1), see Figure 5c), it was indicated by the abrupt gradient change from the current-time curve (see Figure 4).

In Phase 2 of 0.5 mM NaCl → 0.5 mM NaHCO_3_, displacement of the reduced concentration 0.375 mM NaHCO_3_ by 0.5 mM NaCl continues (see Figure 5d). Since 0.5 mM NaCl has higher conductivity than 0.375 mM NaHCO_3_ (see Table 2), an increase in current was detected for Phase 2 (see Figure 4). However, 0.375 mM NaHCO_3_ cannot be completely flushed out, and the normalized X* is unchanged at 0.75 when T* = 1 (see Figure 5d), where X* = *X*/*L* with *X* the axial coordinate and *L* the microchannel length. The gradient of EOF induced a convective flux (term on the right of Equation (6)) experienced by the Cl^−^ anions, which gradually reduces as the flow propagates downstream. This is due to the displacement of NaHCO_3_ out of the microchannel; due to its high pH, the NaHCO_3_ solution has high absolute zeta potential value (see Table 2 and Table 3) that are supposed to facilitate faster EOF. Thus, the gradient of electromigrative flux (third term left of Equation (6)) cancels out that of the convective flux for Cl^−^ anions. This resulted in the interface staying unchanged at X* = 0.75 when T* = 1 (see Figure 5d), and the current stabilized thereafter (see Figure 4). According to the discussion in our previous study [32], for 0.5 mM NaCl → 0.5 mM NaHCO_3_, as a result of the low conductivity of NaHCO_3_ (see Table 2), the residing ion-depletion region (0.375 mM NaHCO_3_) has a higher electric field than 0.5 mM NaCl (displacing electrolyte). Thus, as HCO_3_^−^ ions diffuse to the boundary of the displacing electrolyte, they decelerate due to the low electric field in the displacing electrolyte region. This results in a sharp and constant interfacial region (see Figure 5). 

**Figure 6:**
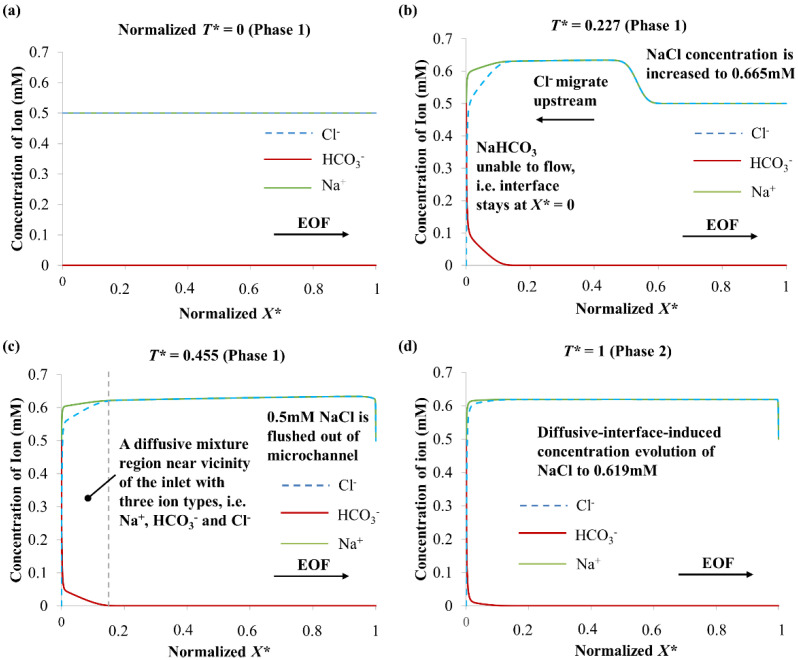
Numerically simulated ion concentrations along the microchannel when 0.5 mM NaHCO_3_ → 0.5 mM NaCl (arrow indicates EOF direction) in (**a**) Phase 1 T* = 0, (**b**) Phase 1 T* = 0.227, (**c**) Phase 1 T* = 0.455 and (**d**) Phase 2 T* = 1. Normalized time T* = *T*/*T^SS^_NaCl_*_→ *NaHCO*3_, where *T^SS^_NaCl_*_→ *NaHCO*3_ is time to reach steady-state current for NaCl → NaHCO_3_. Normalized X* = X/*L*, where *X* is axial coordinate and *L* microchannel length.

The simulated ion concentration distributions when 0.5 mM NaHCO_3_ → 0.5 mM NaCl are shown in Figure 6. The 0.5 mM NaHCO_3_ is unable to flow out from the inlet reservoir to the microchannel for the entire displacement process, i.e., the interface stays at X* = 0. The HCO_3_^−^ anions experience much stronger gradient of electromigrative flux than the EOF induced convective flux. This is because the initial residing solution NaCl has a low absolute zeta potential value (due to its low pH value) (see Table 2 and Table 3) that generates slower EOF.

At T* = 0.227 (Phase 1 of 0.5 mM NaHCO_3_ → 0.5 mM NaCl), accumulation of Na^+^ and Cl^−^ ions occurs, and the increase of NaCl concentration takes place at 0.665 mM (see Figure 6b). The KRF value of 0.5 mM NaHCO_3_ is 2.12 × 10^7^ mol·V·s·m^−5^, and the KRF value of 0.5 mM NaCl is 1.59 × 10^7^ mol·V·s·m^−5^. To match the KRF of 0.5 mM NaHCO_3_, the upstream migration of Cl^−^ causes the NaCl concentration to increase from 0.5 mM to 0.665 mM (solution concentration derived from known KRF of 0.5 mM NaHCO_3_ and ion mobilities of Na^+^ and Cl^−^ from Table 4, see Equation (11)), which leads to an increase in conductivity. Hence, an increase in current was observed for 0.5 mM NaHCO_3_ → 0.5 mM NaCl in Phase 1 (see Figure 4). The residing solution 0.5 mM NaCl was flushed out of the microchannel (at T* = 0.455 (Phase 1), see Figure 6c); this was indicated by the abrupt gradient change from the current-time curve (see Figure 4).

For 0.5 mM NaHCO_3_ → 0.5 mM NaCl in Phase 2, a second concentration evolution happens (see Figure 6d). Initially, the inlet reservoir and the microchannel were filled with 0.5 mM NaHCO_3_ and 0.5 mM NaCl, respectively. With the inlet reservoir acting as an infinite source for the HCO_3_^−^ ions, as well as an infinite sink for the incoming Cl^−^ (migrating upstream), a diffusive mixture region developed near the vicinity of the inlet with three ion types, i.e., Na^+^, HCO_3_^−^, and Cl^−^ (see Figure 6), which fulfill both current continuity and electroneutrality. This diffusive-interface-induced concentration evolution has been discussed in detail in our previous investigation on EOF with dissimilar anion species [33]. The equilibrium concentration after the second evolution in the microchannel can be determined with [33]:

*c_L_* = *I^SS^*_(*Exp*)_/[*_L_E_L_F*∑*n_i_z_i_u_m(i)_*] (12)

where *I^SS^_(Exp)_* is the steady-state experimental current, *E_L_* the applied electric field along the microchannel, *F* the Faraday constant, *A_L_* the channel cross-sectional area, and *n_i_* the molecular formula.

The numerical simulation of 0.5 mM NaHCO_3_ → 0.5 mM NaCl demonstrates that the NaCl concentration decreases from 0.665 mM to 0.619 mM at the end of Phase 2 when T* = 1 (see Figure 6d). Electroosmotic displacement flow of 0.619 mM NaCl and 0.665 mM NaCl progresses during Phase 2. As such, a slight current decrease was observed, and the current stabilized after the displacement process (see Figure 4). Through calculation with Equation (12) based on the experimental data, the NaCl equilibrium concentration is 0.640 ± 0.002 mM, and is reasonably similar to the simulated NaCl equilibrium concentration of 0.619 mM.

**Figure 7:**
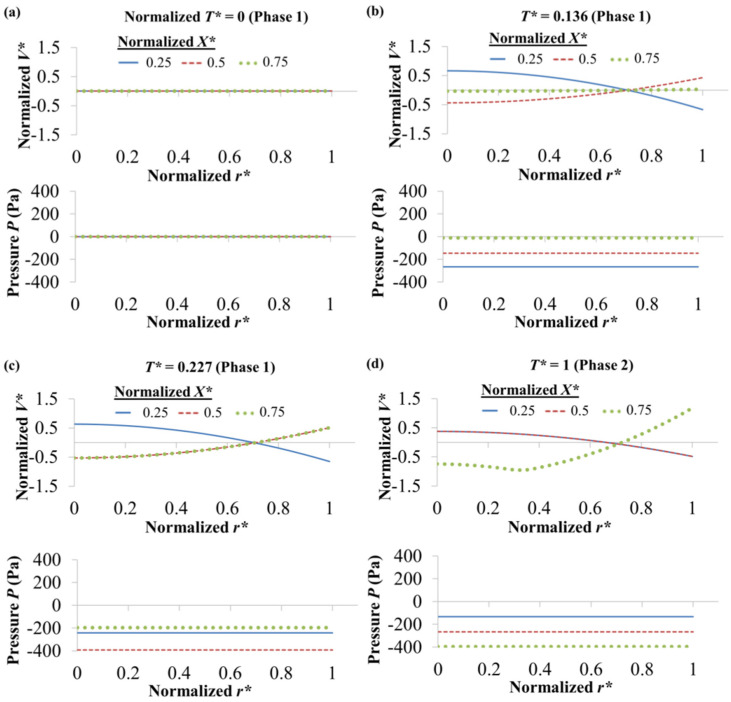
Numerically simulated normalized velocity V* and pressure *P* along normalized radial r* for normalized X* = 0.25, 0.5 and 0.75 of 0.5 mM NaCl → 0.5 mM NaHCO_3_ (arrow indicates EOF direction) in (**a**) Phase 1 T* = 0, (**b**) Phase 1 T* = 0.136, (**c**) Phase 1 T* = 0.227 and (**d**) Phase 2 T* = 1. Normalized V* = (*V* − *V_Avg_*)/*V_Avg_*. Average velocity *V_Avg_* = *Q*/*A_L_* = *Q*/(π*R*^2^), where *Q* is flow rate obtained by integrating radial velocity over microchannel cross sectional area *A_L_*, and *R* is channel radius. Normalized r* = *r*/*R*. Normalized time T* = *T*/*T^SS^_NaCl_*_→ *NaHCO*3_, where *T^SS^_NaCl_*_→ *NaHCO*3_ is time to reach steady-state current for NaCl → NaHCO_3_. Normalized X*= X/*L*, where *X* is axial coordinate and *L* channel length.

**Figure 8:**
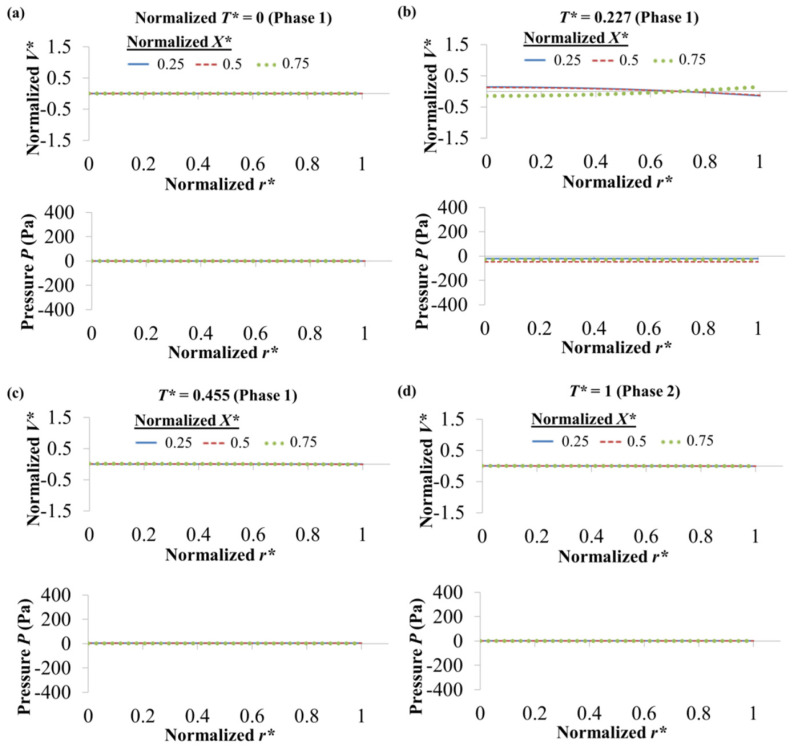
Numerically simulated normalized velocity V* and pressure *P* along normalized radial r* for normalized X* = 0.25, 0.5 and 0.75 of 0.5 mM NaHCO_3_ → 0.5 mM NaCl (arrow indicates EOF direction) in (**a**) Phase 1 T* = 0, (**b**) Phase 1 T* = 0.227, (**c**) Phase 1 T* = 0.455 and (**d**) Phase 2 T* = 1. Normalized V* = (*V* − *V_Avg_*)/*V_Avg_*. Average velocity *V_Avg_* = *Q*/*A_L_* = *Q*/(π*R*^2^), where *Q* is flow rate obtained by integrating radial velocity over microchannel cross sectional area *A_L_*, and *R* is channel radius. Normalized r* = *r*/*R*. Normalized time T* = *T*/*T^SS^_NaCl_*_→ *NaHCO*3_, where *T^SS^_NaCl_*_→ *NaHCO*3_ is time to reach steady-state current for NaCl → NaHCO_3_. Normalized X*= X/*L*, where *X* is axial coordinate and *L* channel length.

To maintain flow continuity over the entire microchannel during the electroosmotic displacement flow process, pseudo-pressure is generated due to non-uniform zeta potential. This resulted velocity profile resembles a combination of electroosmotic flow and pressure driven flow distributions, and deviates from the usual plug-like EOF profile. The simulated normalized velocity V* = (*V* − *V_Avg_*)/*V_Avg_* and pressure *P* along the normalized radial r* = *r*/*R* for normalized X* = 0.25, 0.5, and 0.75 of 0.5 mM NaCl–0.5 mM NaHCO_3_ in two different flow directions are presented in Figure 7 and Figure 8, respectively. The average velocity *V_Avg_* = *Q*/*A_L_* = *Q*/(π*R^2^*), where *Q* is the flow rate obtained by integrating the radial velocity over the channel cross sectional area *A_L_*, and *R* is the channel radius.

For 0.5 mM NaCl → 0.5 mM NaHCO_3_, when normalized time T* = 0, V* and *P* for the different X* values are zero across r*, see Figure 7a. This is because of the uniform zeta potential of the residing NaHCO_3_ at the initial state (see Figure 5a); plug-like EOF profile is observed, with the velocity having zero deviation (i.e., V* = 0) from the average velocity. As the displacement process progresses, as shown in Figure 5b–d, concentration evolutions occur with different fluid segments along the microchannel having different and thus non-uniform zeta potential; this generates different wall driving force. To maintain fluid flow continuity, internal fluid pressure is generated, which changes along the axial length. Negative pressure (back pressure) is generated due to EOF slowing down (reduction of velocity) as the displacement flow progresses. This fluid pressure variation causes the flow velocity profiles to evolve and deviate from the plug-like EOF profile, as shown in Figure 7b–d. For 0.5 mM NaHCO_3_ → 0.5 mM NaCl, when T* = 0, V*, and *P* for the different X* values are zero across r* (see Figure 8a) due to the uniform zeta potential of the residing NaCl at the initial state (see Figure 6a). However, NaHCO_3_ is unable to flow out from the inlet, and only slight concentration evolutions are shown in Figure 6b-d. Therefore, the pressure does not vary significant, and only small deviations of the flow velocity profile from the plug-like EOF profile are observed in Figure 8b–d.

**Figure 9:**
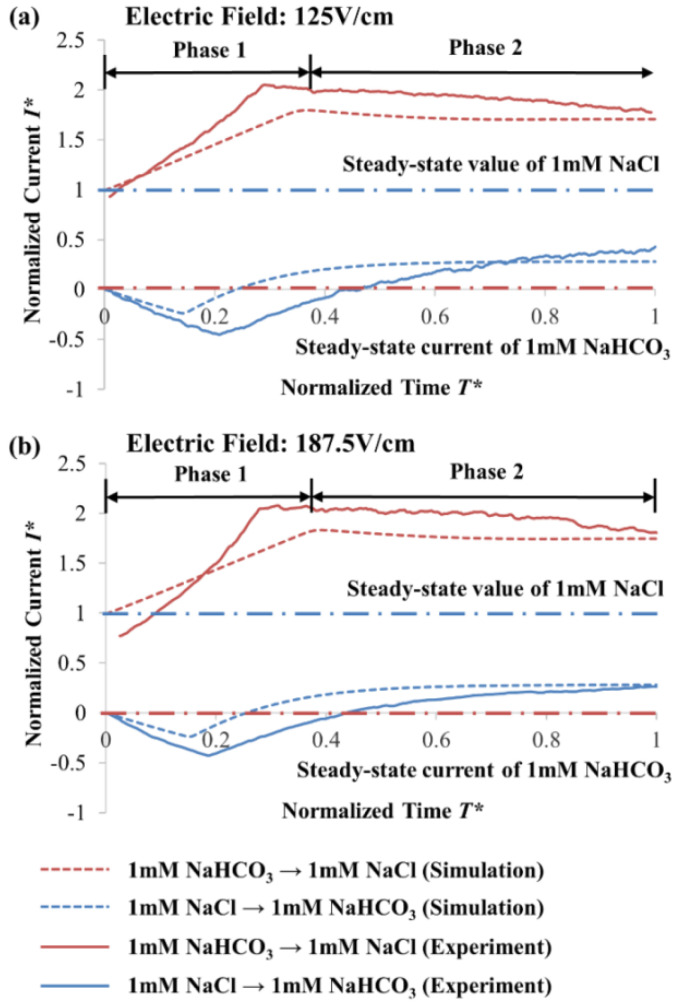
Electroosmotic displacement flow of the 1mM NaCl–NaHCO_3_ solution pair at applied electric field of (**a**) 125 V·cm^−1^ and (**b**) 187.5 V·cm^−1^, whereby numerical simulation and experimental results are compared. Normalized currents and times are calculated with I* = (*I* − *I*^0^_*NaHCO*3_)/(*I*^0^_*NaCl*_ − *I*^0^_*NaHCO*3_) and T* = *T*/*T^SS^_NaCl_*_→ *NaHCO*3_ respectively, where initial currents of solutions are represented by *I*^0^ with *NaHC*O_3_ and *NaCl* subscripts respectively, and the time to reach steady-state current for NaCl → NaHCO_3_ (arrow indicates EOF direction) is represented by *T^SS^_NaCl_*_→ *NaHCO*3_.

The effect of varying the concentration of the solution pair was examined with 1 mM NaHCO_3_–NaCl solution pair (see Figure 9a). The electric field was kept the same as the case for the 0.5 mM NaHCO_3_–NaCl solution pair, in which the electric field strength was 125 V·cm^−1^. The numerically predicted results match well with the experimental observations (see Figure 9a). The overall trend is almost identical to 0.5 mM NaHCO_3_–NaCl (see Figure 4) with an abrupt gradient change observed (separating Phases 1 and 2), and the current-time curve was unable to reach the current of the displacing solution. For 1 mM NaCl → 1 mM NaHCO_3_, the NaHCO_3_ concentration is reduced to 0.750 mM (according to Kohlrausch regulating function (KRF), see Equation (11)), which is two times that of 0.5 mM NaCl displacing 0.5 mM NaHCO_3_. Supposedly, the difference in concentration should affect the zeta potential and hence EOF strength. However, interestingly, the competing effect between the solution concentration and pH results in insignificant variation of the absolute zeta potential value, which can be seen from Table 2 and Table 3. Hence, the displacement interface of 0.750 mM NaHCO_3_ stays unchanged at normalized X* = 0.75. For 1 mM NaHCO_3_ → 1 mM NaCl, 1 mM NaHCO_3_ cannot be displaced into the microchannel. The diffusive-interface-induced evolution near the inlet vicinity results in NaCl equilibrium concentration of 1.21 ± 0.012 mM (calculated based on experimental data with Equation (12)), which is approximately two times that of 0.5 mM NaHCO_3_ displacing 0.5 mM NaCl.

To study its effect on EOF with the 1 mM NaHCO_3_–NaCl solution pair, the electric field was increased by 50% to 187.5 V·cm^−1^, as shown in Figure 9b. Good agreement is obtained between simulations and experimental results. The overall trend follows closely, and is similar to that of 1 mM NaHCO_3_–NaCl with an electric field of 125 V·cm^−1^ (see Figure 9a) and 0.5 mM NaHCO_3_–NaCl with an electric field of 125 V·cm^−1^ (see Figure 4). For 1 mM NaCl → 1 mM NaHCO_3_, despite increasing the electric field by 50% to 187.5 V·cm^−1^, the NaHCO_3_ concentration is similarly reduced to 0.750 mM (not affected based on KRF, see Equation (11)) and without complete displacement by 1 mM NaCl. For 1 mM NaHCO_3_ → 1 mM NaCl with an electric field of 187.5 V·cm^−1^, the NaCl equilibrium concentration is calculated to be 1.16 ± 0.050 mM (based on experimental data with Equation (12)), which is approximately the same as the case with the electric field of 125 V·cm^−1^. Changing the electric field strength has no influence on the concentration evolution for the NaHCO_3_–NaCl solution pair system, except producing a much faster electroosmotic displacement process.

**Figure 10:**
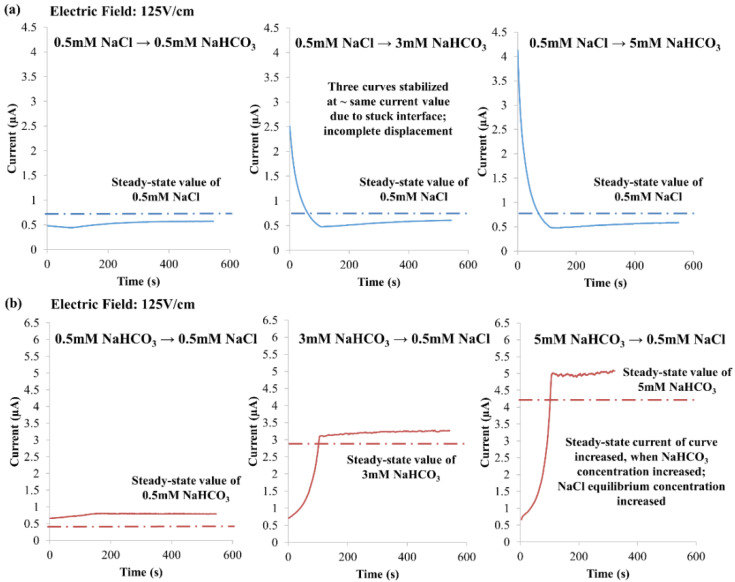
Experimental observations of varying NaHCO_3_ concentration, i.e., 0.5 mM, 3 mM and 5 mM, with the NaCl concentration fixed at 0.5 mM and an electric field of 125 V·cm^−1^. (**a**) NaCl → NaHCO_3_ (arrow indicates EOF direction), and (**b**) NaHCO_3_ → NaCl.

The effect of varying NaHCO_3_ concentration was investigated experimentally by employing 0.5 mM, 3 mM or 5 mM NaHCO_3_, with the NaCl concentration fixed at 0.5 mM and an electric field of 125 V·cm^−1^, as shown in Figure 10. For NaCl → NaHCO_3_, the NaHCO_3_ concentration is reduced to 0.375 mM for different NaHCO_3_ concentrations with 0.5 mM NaCl (according to Kohlrausch regulating function (KRF), see Equation (11)). With the displacement of 0.375 mM NaHCO_3_ stayed unchanged at normalized interface X* = 0.75, the current-time curves stabilized at approximately the same current value despite different NaHCO_3_ concentrations, as shown in Figure 10a. For NaHCO_3_ → NaCl, NaHCO_3_ still cannot displace into the microchannel. The diffusive-interface-induced evolution near the inlet vicinity results in NaCl equilibrium concentrations for 0.5 mM, 3 mM and 5 mM NaHCO_3_ to be 0.640 ± 0.002 mM, 2.59 ± 0.02 mM and 4.02 ± 0.04 mM, respectively (calculated based on experimental data with Equation (12)). The increase in the NaCl equilibrium concentration was captured by the increase in the steady-state current of the current-time curve, when NaHCO_3_ concentration was increased, see Figure 10b.

**Figure 11:**
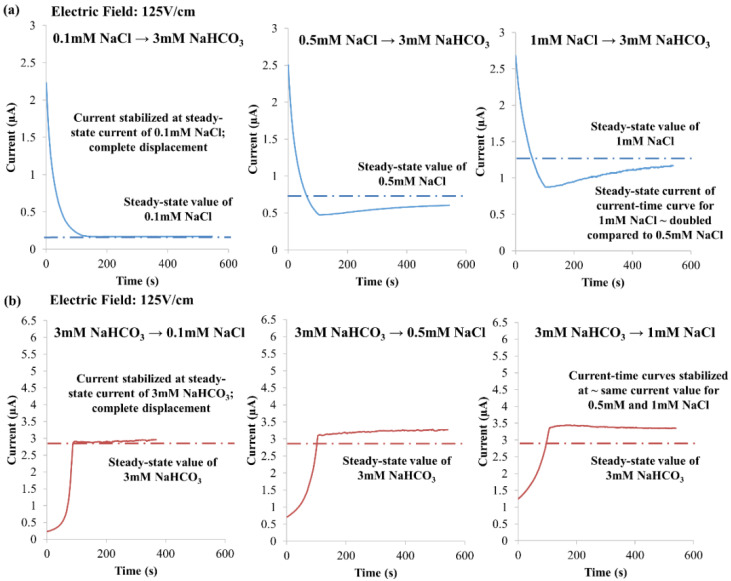
Experimental observations of varying NaCl concentration, i.e., 0.1 mM, 0.5 mM and 1 mM, with the NaHCO_3_ concentration fixed at 3 mM and an electric field of 125 V·cm^−1^. (**a**) NaCl → NaHCO_3_ (arrow indicates EOF direction), and (**b**) NaHCO_3_ → NaCl.

Investigation on the influence of varying NaCl concentration was experimentally conducted with 0.1 mM, 0.5 mM and 1 mM NaCl, where the NaHCO_3_ concentration was fixed at 3 mM and electric field at 125 V·cm^−1^, see Figure 11. For NaCl → NaHCO_3_, the NaHCO_3_ concentrations for 0.5 mM and 1 mM NaCl reduce to 0.375 mM and 0.750 mM, respectively (according to KRF, see Equation (11)). As such, the steady-state current of the current-time curve was approximately doubled for 1 mM NaCl, as compared to 0.5 mM NaCl, see Figure 11a. However, complete displacement was realized when 0.1 mM NaCl was employed to displace 3 mM NaHCO_3_, with the current stabilized at the steady-state current of 0.1 mM NaCl (see Figure 11a). For NaHCO_3_ → NaCl, the NaCl equilibrium concentrations for 0.5 mM and 1 mM NaCl are 2.59 ± 0.02 mM and 2.70 ± 0.01 mM, respectively (calculated based on experimental data with Equation (12)). Since the NaHCO_3_ concentration was the same (fixed at 3 mM), the current-time curves stabilized at approximately the same current value for 0.5 mM and 1 mM NaCl, as shown in Figure 11b. While NaHCO_3_ completely displaced NaCl (with a concentration of 0.1 mM), the current stabilized at the steady-state current of 3 mM NaHCO_3_, see Figure 11b. Through lowering the NaCl concentration, the absolute zeta potential is increased, see Table 3; this enables stronger EOF for the displacement process.

## 5. Conclusions

Electroosmotic displacement flow involving multiple fluids exhibits EOF hysteresis, i.e., flow direction-dependent behavior. Thus far, no study has been conducted on the hysteresis effect for a solution pair system comprising of dissimilar ionic species and substantial pH difference; their rather different characteristics tend to result in a complex hysteretic behavior. In this investigation, the NaHCO_3_–NaCl solution pair was chosen as a model system to examine the hysteresis phenomenon.

The EOF of the NaHCO_3_–NaCl solution pair was carried out through current monitoring experiments. Finite element numerical simulations based on slip velocity model with modified wall boundary condition were performed for the evaluation of the displacement flow behaviors and ion concentration distributions. Quantitative agreements were achieved between experimental and simulation results.

For NaCl → NaHCO_3_ (the arrow indicates EOF direction), a concentration evolution of NaHCO_3_ happens. The displacement of the original residing and evolved NaHCO_3_ concentrations with high absolute zeta potential values (due to high pH values) by NaCl with low absolute zeta potential value causes the EOF to be reduced. As a result, NaCl is not fully displaced within the microchannel due to the gradient of the electromigrative flux cancelling that of the convective flux. 

For NaHCO_3_ → NaHCO_3_, NaHCO_3_ cannot displace into the microchannel. This rather surprising and counter-intuitive outcome is a result of the stronger gradient of electromigrative flux than convective flux, as NaCl has low absolute zeta potential value (due to a low pH value) that generates slow EOF. Hence, evolution of the diffusive-interface-induced concentration of NaCl occurs.

The aforesaid flow characteristics are independent of the applied electric field. However, through lowering the NaCl concentration, the absolute zeta potential value is increased, which enables EOF to increase for achieving complete displacement. While varying the NaHCO_3_ concentration has negligible impact on the displacement process.

The outcomes of this investigation could provide a proper understanding of the flow behavior of inhomogeneous solutions with dissimilar pH and ion species for micro-/nanofluidic applications, such as isotachophoresis (ITP) and field amplified sample stacking (FASS).

## Figures and Tables

**Table 1:** Experimental conditions with parameter variations for electroosmotic displacement flow of sodium bicarbonate (NaHCO_3_) and sodium chloride (NaCl) solution pair in two flow directions, i.e., NaCl → NaHCO_3_ (arrow indicates EOF direction), and NaHCO_3_ → NaCl.

Parameters Investigated	Experimental Conditions with Parameter Variations (Two Flow Directions)	
Concentration of solution pair	0.5 mM NaHCO_3_–0.5 mM NaCl Electric field = 125 V·cm^−1^	1 mM NaHCO_3_–1 mM NaCl Electric field = 125 V·cm^−1^
Applied electric field	1 mM NaHCO_3_–1 mM NaCl Electric field = 125 V·cm^−1^	1 mM NaHCO_3_–1 mM NaCl Electric field = 187.5 V·cm^−1^
Concentration of individual solution	0.5 mM NaHCO_3_–0.5 mM NaCl 3 mM NaHCO_3_–0.5 mM NaCl 5 mM NaHCO_3_–0.5 mM NaCl Electric field = 125 V·cm^−1^	3 mM NaHCO_3_–0.1 mM NaCl 3 mM NaHCO_3_–0.5 mM NaCl 3 mM NaHCO_3_–1 mM NaCl Electric field = 125 V·cm^−1^

**Table 2:** Measured pH values and conductivities for experiments.

Solutions	pH	Conductivities (µS·cm^−1^)
0.1 mM NaCl	5.53 ± 0.02	18.8 ± 0.1
0.5 mM NaCl	5.68 ± 0.03	63.5 ± 0.1
0.619 mM NaCl	5.49 ± 0.05	76.2 ± 0.2
1 mM NaCl	5.64 ± 0.02	127.6 ± 0.2
1.24 mM NaCl	5.88 ± 0.01	186.6 ± 0.3
0.375 mM NaHCO_3_	7.35 ± 0.03	38.1 ± 0.2
0.5 mM NaHCO_3_	7.62 ± 0.03	46.1 ± 0.2
0.750 mM NaHCO_3_	7.82 ± 0.01	76.3 ± 0.1
1 mM NaHCO_3_	7.90 ± 0.02	93.8 ± 0.3
3 mM NaHCO_3_	8.17 ± 0.01	276.3 ± 0.4
5 mM NaHCO_3_	8.69 ± 0.01	466.6 ± 0.2

**Table 3:** Measured zeta potential values of solutions for numerical simulations.

Solutions	Zeta Potential (mV)
0.5 mM NaCl	−54.3 ± 1.5
0.619 mM NaCl	−51.5 ± 3.0
1 mM NaCl	−47.8 ± 2.3
1.24 mM NaCl	−39.8 ± 1.6
0.375 mM NaHCO_3_	−128 ± 8.8
0.5 mM NaHCO_3_	−122 ± 12.7
0.750 mM NaHCO_3_	−121 ± 6.4
1 mM NaHCO_3_	−117 ± 3.8

**Table 4:** Symbols and values of constants for simulations, with ion mobility determined by (*z_i_D_i_F*)/(*RT*).

Constants	Symbol (Unit)	Value
Fluid relative permittivity	*ε_r_*	80
Free space permittivity	*ε_o_* (C·V^−1^·m^−1^)	8.85 × 10^−12^
Fluid density	*ρ* (kg·m^−3^)	1000
Fluid viscosity	*µ* (kg·m^−1^·s^−1^)	8.90 × 10^−4^
Faraday constant	*F* (C·mol^−1^)	96,485
Boltzmann constant	*k_b_* (m^2^·kg·s^−2^·K^−1^)	1.381 × 10^−23^
Gas constant	*R* (J·mol^−1^·K^−1^)	8.314
Temperature	*T* (K)	298
Boltzmann constant	*k_b_* (m^2^·kg·s^−2^·K^−1^)	1.381 × 10^−23^
Avogadro constant	*N_a_* (mol^−1^)	6.022 × 10^23^
Electron charge	*e* (C)	1.602 × 10^−19^
Na^+^ diffusion coefficient	*D_Na_^+^* (m^2^·s^−1^)	1.334 × 10^−9^ [50]
Cl^−^ diffusion coefficient	*D_Cl_*^−^ (m^2^·s^−1^)	2.032 × 10^−9^ [50]
HCO_3_^−^ diffusion coefficient	*D_HCO_*_3_^−^ (m^2^·s^−1^)	1.105 × 10^−9^ [50]
Na^+^ mobility	*u_m(Na_^+^_)_* (m^2^·V^−1^·s^−1^)	5.194 × 10^−8^
Cl^−^ mobility	*u_m(Cl_*^−^*_)_* (m^2^·V^−1^·s^−1^)	−7.919 × 10^−8^
HCO_3_^−^ mobility	*u_m(HCO_*_3_^−^*_)_* (m^2^·V^−1^·s^−1^)	−4.303 × 10^−8^
Na^+^ charge number	*z_Na_^+^*	+1
Cl^−^ charge number	*z_Cl_* ^−^	−1
HCO_3_^−^ charge number	*z_HCO_*_3_^−^	−1

**Table 5:** Boundary conditions for the steady-state single fluid EOF numerical model.

Variable	Condition ^a^	Boundary
Applied potential *φ*	*φ* = 62.5 V/93.75 V	Inlet
	*φ* = 0 V	Outlet
	−**n**·*σ*∇*φ* = 0	Wall and symmetry
Concentrations of ions *c_i_*	*c_i_* of residing solution	Inlet and outlet
	−**n**·[−*D_i_*∇*c_i_ – u_m(i)_c_i_*∇*φ* + *vc_i_*] = 0	Wall and symmetry
Flow velocity *v* and Pressure *p*	*v* = *ε_r_ε_o_ζ_Function_*∇*φ*/*µ*	Wall
	*p* = 0	Inlet and outlet

^a^ where **n** is unit vector normal to boundary, *ε_r_* fluid relative permittivity, *ε_o_* free space permittivity, *c_i_* ion concentration, *σ* solution conductivity, *u_m(i)_* ion mobility, *D_i_* ion diffusion coefficient, and *ζ_Function_* varying wall zeta potential function.

**Table 6:** Evolved concentrations for different EOF directions obtained via simulations. Arrow indicates EOF direction.

EOF Directions	Evolved Concentrations
0.5 mM NaCl → 0.5 mM NaHCO_3_	0.375 mM NaHCO_3_
0.5 mM NaHCO_3_ → 0.5 mM NaCl	0.619 mM NaCl
1 mM NaCl → 1 mM NaHCO_3_	0.750 mM NaHCO_3_
1 mM NaHCO_3_ → 1 mM NaCl	1.24 mM NaCl

**Table 7:** Varying wall zeta potential functions for different EOF directions. Arrow indicates EOF direction.

EOF Directions	Varying Wall Zeta Potential Functions ^a^, *ζ_Function_*
0.5 mM NaCl → 0.5 mM NaHCO_3_	[(*ζ*_0.5mM *NaHCO*3_ − *ζ*_0.375mM *NaHCO*3_) * flc1hs(*c_Na_^+^* − 0.44, 0.05) + *ζ*_0.375mM *NaHCO*3_] − [(*ζ*_0.5mM *NaHCO*3_ − *ζ*_0.5 mM *NaCl*_) * flc1hs(*c_Cl_^−^* − 0.25, 0.05)]
0.5 mM NaHCO_3_ → 0.5 mM NaCl	[(*ζ*_0.619mM *NaCl*_ − *ζ*_0.5mM *NaCl*_) * flc1hs(*c_Na_^+^* − 0.56, 0.05) + *ζ*_0.5mM *NaCl*_] − [(*ζ*_0.5mM *NaCl*_ − *ζ*_0.5mM *NaHCO*3_) * flc1hs(*c*_*HCO*3_^−^ − 0.25, 0.05)]
1 mM NaCl → 1 mM NaHCO_3_	[(*ζ*_1mM *NaHCO*3_ − *ζ*_0.750mM *NaHCO*3_) * flc1hs(*c_Na_^+^* − 0.88, 0.05) + *ζ*_0.750mM *NaHCO*3_] − [(*ζ*_1mM *NaHCO*3_ − *ζ*_1mM *NaCl*_) * flc1hs(*c_Cl_*^−^ − 0.5, 0.05)]
1 mM NaHCO_3_ → 1 mM NaCl	[(*ζ*_1.24mM *NaCl*_ − *ζ*_1mM *NaCl*_) * flc1hs(*c_Na_^+^* − 1.15, 0.05) + *ζ*_1mM *NaCl*_] − [(*ζ*_1mM *NaCl*_ − *ζ*_1mM *NaHCO*3_) * flc1hs(*c*_*HCO*3_^−^ − 0.5, 0.05)]

^a^ where flc1hs represents a smoothed step function (or Heaviside function), *c_i_* is ion concentration, ζ with *NaHCO*_3_ and *NaCl solution concentrations* subscripts represent the experimentally measured zeta potentials of solutions (see Table 3).

## References

[1] Erickson D., Liu X., Krull U., Li D. (2004). Electrokinetically controlled DNA hybridization microfluidic chip enabling rapid target analysis. Anal. Chem..

[2] Weng X., Jiang H., Chon C.H., Chen S., Cao H., Li D. (2011). An RNA–DNA hybridization assay chip with electrokinetically controlled oil droplet valves for sequential microfluidic operations. J. Biotechnol..

[3] Xiong Q., Lim A.E., Lim Y., Lam Y.C., Duan H. (2019). Dynamic magnetic nanomixers for improved microarray assays by eliminating diffusion limitation. Adv. Healthc. Mater..

[4] Oddy M., Santiago J.G., Mikkelsen J. (2001). Electrokinetic instability micromixing. Anal. Chem..

[5] Balasuriya S. (2015). Dynamical systems techniques for enhancing microfluidic mixing. J. Micromech. Microeng..

[6] Yao S., Hertzog D.E., Zeng S., Mikkelsen J.C., Santiago J.G. (2015). Porous glass electroosmotic pumps: Design and experiments. J. Colloid Interface Sci..

[7] Buie C.R., Posner J.D., Fabian T., Cha S.W., Kim D., Prinz F.B., Eaton J.K., Santiago J.G. (2006). Water management in proton exchange membrane fuel cells using integrated electroosmotic pumping. J. Power Sources.

[8] Liu M., Liu Y., Guo Q., Yang J. (2009). Modeling of electroosmotic pumping of nonconducting liquids and biofluids by a two-phase flow method. J. Electroanal. Chem..

[9] Lim A.E., Lim C.Y., Lam Y.C., Lim Y.H. (2019). Effect of microchannel junction angle on two-phase liquid-gas Taylor flow. Chem. Eng. Sci..

[10] Kawamata T., Yamada M., Yasuda M., Seki M. (2008). Continuous and precise particle separation by electroosmotic flow control in microfluidic devices. Electrophoresis.

[11] Hua Y., Jemere A.B., Dragoljic J., Harrison D.J. (2013). Multiplexed electrokinetic sample fractionation, preconcentration and elution for proteomics. Lab Chip.

[12] Giordano B.C., Burgi D.S., Hart S.J., Terray A. (2012). On-line sample pre-concentration in microfluidic devices: A review. Anal. Chim. Acta.

[13] Delmotte P. (1979). Capillary isotachophoresis. J. Chromatogr. A.

[14] Garcia-Schwarz G., Bercovici M., Marshall L.A., Santiago J.G. (2011). Sample dispersion in isotachophoresis. J. Fluid Mech..

[15] Smejkal P., Bottenus D., Breadmore M.C., Guijt R.M., Ivory C.F., Foret F., Macka M. (2013). Microfluidic isotachophoresis: A review. Electrophoresis.

[16] Bharadwaj R., Santiago J.G. (2005). Dynamics of field-amplified sample stacking. J. Fluid Mech..

[17] Sustarich J.M., Storey B.D., Pennathur S. (2010). Field-amplified sample stacking and focusing in nanofluidic channels. Phys. Fluids.

[18] Horvath J., Dolník V. (2001). Polymer wall coatings for capillary electrophoresis. Electrophoresis.

[19] Preisler J., Yeung E.S. (1996). Characterization of nonbonded poly (ethylene oxide) coating for capillary electrophoresis via continuous monitoring of electroosmotic flow. Anal. Chem..

[20] Lim A.E., Lim C.Y., Lam Y.C., Taboryski R., Wang S.R. (2017). Effect of nanostructures orientation on electroosmotic flow in a microfluidic channel. Nanotechnology.

[21] Lim A.E., Lim C.Y., Lam Y.C., Taboryski R. (2018). Electroosmotic flow in microchannel with black silicon nanostructures. Micromachines.

[22] Lim A.E., Lam Y.C. (2020). Numerical investigation of nanostructure orientation on electroosmotic flow. Micromachines.

[23] Tang S.W., Chang C.H., Wei H.H. (2011). Roles of solution conductivity mismatch in transient current and fluid transport in electrolyte displacement by electro-osmotic flow. Microfluid. Nanofluid..

[24] Ren L., Masliyah J., Li D. (2003). Experimental and theoretical study of the displacement process between two electrolyte solutions in a microchannel. J. Colloid Interface Sci..

[25] Ren L., Escobedo C., Li D. (2001). Electroosmotic flow in a microcapillary with one solution displacing another solution. J. Colloid Interface Sci..

[26] Mampallil D., van den Ende D., Mugele F. (2010). A simple method to determine the surface charge in microfluidic channels. Electrophoresis.

[27] Leong I.W., Tsutsui M., Murayama S., He Y., Taniguchi M. (2020). Electroosmosis-Driven Nanofluidic Diodes. J. Phys. Chem. B.

[28] Zeng Z., Ai Y., Qian S. (2014). pH-regulated ionic current rectification in conical nanopores functionalized with polyelectrolyte brushes. Phys. Chem. Chem. Phys..

[29] Kusama S., Sato K., Matsui Y., Kimura N., Abe H., Yoshida S., Nishizawa M. (2021). Transdermal electroosmotic flow generated by a porous microneedle array patch. Nat. Commun..

[30] Lim C.Y., Lim A.E., Lam Y.C. (2016). Ionic origin of electro-osmotic flow hysteresis. Sci. Rep..

[31] Lim C.Y., Lim A.E., Lam Y.C. (2017). pH change in electroosmotic flow hysteresis. Anal. Chem..

[32] Lim A.E., Lim C.Y., Lam Y.C. (2015). Electroosmotic flow hysteresis for dissimilar ionic solutions. Biomicrofluidics.

[33] Lim A.E., Lim C.Y., Lam Y.C. (2016). Electroosmotic flow hysteresis for dissimilar anionic solutions. Anal. Chem..

[34] Zhang L., McNeece C.J., Hesse M.A., Wang M. (2018). Reactive transport of protons in electro-osmotic displacements with electrolyte concentration difference in a microcapillary. Anal. Chem..

[35] McCreedy T. (2000). Fabrication techniques and materials commonly used for the production of microreactors and micro total analytical systems. Trends Anal. Chem..

[36] Soga T., Inoue Y., Ross G.A. (1995). Analysis of halides, oxyhalides and metal oxoacids by capillary electrophoresis with suppressed electroosmotic flow. J. Chromatogr. A.

[37] Nelstrop L.J., Greenwood P.A., Greenway G.M. (2001). An investigation of electroosmotic flow and pressure pumped luminol chemiluminescence detection for cobalt analysis in a miniaturised total analytical system. Lab Chip.

[38] Yan D.G., Yang C., Huang X.Y. (2007). Effect of finite reservoir size on electroosmotic flow in microchannels. Microfluid. Nanofluid..

[39] Rodríguez I., Chandrasekhar N. (2005). Experimental study and numerical estimation of current changes in electroosmotically pumped microfluidic devices. Electrophoresis.

[40] Almutairi Z.A., Glawdel T., Ren C.L., Johnson D.A. (2009). A Y-channel design for improving zeta potential and surface conductivity measurements using the current monitoring method. Microfluid. Nanofluid..

[41] Tang G., Yan D., Yang C., Gong H., Chai J.C., Lam Y.C. (2006). Assessment of Joule heating and its effects on electroosmotic flow and electrophoretic transport of solutes in microfluidic channels. Electrophoresis.

[42] Arulanandam S., Li D. (2002). Determining ζ potential and surface conductance by monitoring the current in electro-osmotic flow. J. Colloid Interface Sci..

[43] Celebi A.T., Beskok A. (2018). Molecular and continuum transport perspectives on electroosmotic slip flows. J. Phys. Chem. C.

[44] Craven T.J., Rees J.M., Zimmerman W.B. (2008). On slip velocity boundary conditions for electroosmotic flow near sharp corners. Phys. Fluids.

[45] Yan D., Yang C., Miao J., Lam Y., Huang X. (2009). Enhancement of electrokinetically driven microfluidic T-mixer using frequency modulated electric field and channel geometry effects. Electrophoresis.

[46] Zimmerman W.B., Rees J.M., Craven T.J. (2006). Rheometry of non-Newtonian electrokinetic flow in a microchannel T-junction. Microfluid. Nanofluid..

[47] Tang G.Y., Yang C., Chai J.C., Gong H.Q. (2004). Joule heating effect on electroosmotic flow and mass species transport in a microcapillary. Int. J. Heat Mass Transf..

[48] Keh H.J., Tseng H.C. (2001). Transient electrokinetic flow in fine capillaries. J. Colloid Interface Sci..

[49] Yeh L.H., Xue S., Joo S.W., Qian S., Hsu J.P. (2012). Field effect control of surface charge property and electroosmotic flow in nanofluidics. J. Phys. Chem. C.

[50] Newman J., Thomas-Alyea K.E. (2012). Electrochemical Systems.

[51] Hruška V., Gaš B. (2007). Kohlrausch regulating function and other conservation laws in electrophoresis. Electrophoresis.

